# AtPIN: *Arabidopsis thaliana *Protein Interaction Network

**DOI:** 10.1186/1471-2105-10-454

**Published:** 2009-12-31

**Authors:** Marcelo M Brandão, Luiza L Dantas, Marcio C Silva-Filho

**Affiliations:** 1 Departamento de Genética, Escola Superior de Agricultura Luiz de Queiroz, Universidade de São Paulo, Av. Pádua Dias, 11, C.P. 83, 13400-970 Piracicaba, SP, Brazil

## Abstract

**Background:**

Protein-protein interactions (PPIs) constitute one of the most crucial conditions to sustain life in living organisms. To study PPI in Arabidopsis thaliana we have developed AtPIN, a database and web interface for searching and building interaction networks based on publicly available protein-protein interaction datasets.

**Description:**

All interactions were divided into experimentally demonstrated or predicted. The PPIs in the AtPIN database present a cellular compartment classification (C^3^) which divides the PPI into 4 classes according to its interaction evidence and subcellular localization. It has been shown in the literature that a pair of genuine interacting proteins are generally expected to have a common cellular role and proteins that have common interaction partners have a high chance of sharing a common function. In AtPIN, due to its integrative profile, the reliability index for a reported PPI can be postulated in terms of the proportion of interaction partners that two proteins have in common. For this, we implement the Functional Similarity Weight (FSW) calculation for all first level interactions present in AtPIN database. In order to identify target proteins of cytosolic glutamyl-tRNA synthetase (Cyt-gluRS) (AT5G26710) we combined two approaches, AtPIN search and yeast two-hybrid screening. Interestingly, the proteins glutamine synthetase (AT5G35630), a disease resistance protein (AT3G50950) and a zinc finger protein (AT5G24930), which has been predicted as target proteins for Cyt-gluRS by AtPIN, were also detected in the experimental screening.

**Conclusions:**

AtPIN is a friendly and easy-to-use tool that aggregates information on *Arabidopsis thaliana *PPIs, ontology, and sub-cellular localization, and might be a useful and reliable strategy to map protein-protein interactions in Arabidopsis. AtPIN can be accessed at http://bioinfo.esalq.usp.br/atpin.

## Background

Protein-protein interactions (PPIs) constitute one of the most crucial conditions to sustain life in living organisms. Recently, many experimental procedures have been developed to help elucidate the intricate networks of PPIs ranging from high-throughput experiments based on genomic scale analyses [1-4] to molecular biology approaches on a specific key pathway [5-7]. Sometimes the costs (financial and personal) of such exploratory experimental approaches are prohibitive; to circumvent this drawback, the bioinformatics alternative is frequently used as a valuable preliminary step to point to a more specific target, reducing both costs and time.

All of the protein-protein interaction information is often made freely available on different public databases with searching tools commonly restricted to one specific data set. However, even using standard formats to exchange data such as Molecular Interaction XML Format (PSI MI XML))[8] protein nomenclature may differ, impairing comparisons among databases without some protein name conversion.

Some authors make use of methodologies such as yeast two-hybrid, mass spectrometry, immunoprecipitation, or fluorescence resonance energy transfer assays to demonstrate protein interactions [9-14]. But, in some cases, protein interaction networks were determined solely by bioinformatics tools [15-18], and were not confirmed by experimental methodologies. In addition, those predictions rarely consider the subcellular localization of the interactors. The function of a protein is governed by its interaction with other proteins inside a cell, but even if two proteins are consistently predicted to interact they must be located at the same cell compartment and at the same time.

*Arabidopsis thaliana *has long been used as a model organism in a wide range of protein function, interactions and mutational studies [19]. Thus, a lot of predicted and curated data is now available on centralized databanks such as TAIR [20] or throughout scientific literature. In this work, we present the *Arabidopsis thaliana *Protein Interaction Network (AtPIN), a database that integrates five available interaction data sets and two other databases: SUBA, a subcellular localization database [21,22] and TAIR gene ontology and annotation [23]. We also generated a web interface to query AtPIN and built the networks in a Cytoscape [24] easily importing format (XGMML and SIF).

One of the AtPIN key points is its integrative profile, queries response encompass experimental and predicted information on the protein interactions as the subcellular location and its database structure flexibility, facilitating the addition of new data sets, as well as additional analyses parameters. AtPIN presents some advantages upon other available systems: it is specific for *A. thaliana *protein interaction; the scoring system for co-localization; easily integration with Medusa [25] and Cytoscape [24] for PPI network visualization and manipulation.

## Construction and content

### AtPIN database (AtPINDB)

We used MySQL http://www.mysql.com/ to build AtPINDB due to its transactional SQL database engine and fastness. AtPINDB integrates more than 96,000 PPIs (96,221 as in release 8) from five public available databases: IntAct [26,27], BioGRID [28], Arabidopsis protein-protein interaction data curated from the literature by TAIR curators [20,29], the Predicted Interactome for *Arabidopsis *[16], and the *A. thaliana *Protein Interactome Database (AtPID) [30], all of them are queried weekly for updates.

The PPIs demonstration methodologies on AtPINDB were divided into two categories: **Experimental: **This means that the indicated PPI was experimentally demonstrated using *Arabidopsis thaliana *proteins. **Predicted: **The indicated PPI was proposed based on ortholog studies.

### AtPINDB updates

All interaction updates are locally curated, manually and automatically via a homemade set of PERL scripts and performed as follows: 1) If necessary, change the protein identification to TAIR locus name, based on conversion data available at the TAIR website ftp://ftp.arabidopsis.org/home/tair/Proteins/Id_conversions/; 2) update all annotation and gene ontology information to the most current available at TAIR ftp://ftp.arabidopsis.org/home/tair/Ontologies/Gene_Ontology/. 3) update the subcellular information for each locus based on SUBA [21]. 4) update all interactions from databases. Experimentally demonstrated interactions have priority over predicted ones, and once the PPI status is updated its Pubmed links will now represent the direct evidence publication as well as the experimental method used to demonstrate this interaction. 5) Check and update the experiment controlled vocabulary. All experimental data is present in a controlled vocabulary based on the Molecular Interactions from Proteomics Standards Initiative (PSI_MI))[8] available at http://www.berkeleybop.org/ontologies/obo-all/psi-mi/. 6) Recalculate the cellular compartment classification and FSW as described below.

### Cellular Compartment Classification

The cellular compartment classification (C^3 ^value) is represented as classes and is calculated using simple mathematical parameters: type of interaction + co-localization + determination of subcellular localization (experimentally or predicted). The value attributed for the type of interaction is 4 if it is based on experimental data, and 0 if there is no experimental data available (predicted); for co-localization we attribute score 2, otherwise we display score 0; If subcellular localization is based on experimental analyses we score 1, and 0 if predicted. Considering all possibilities we divided the PPIs in the AtPINDB into 5 classes: **Class A **(C^3 ^= 7): The PPI and subcellular location have been shown to be experimentally demonstrated and both proteins are co-localized. **Class B **(C^3 ^= 5): The PPI and subcellular location have been experimentally shown, however, the proteins were localized to different subcellular compartments. **Class C **(C^3 ^= 3): Same as Class A but the PPI is based on prediction analyses. **Class D: **Same as Class A but subcellular location is based on prediction analyses. For this class the same mathematical methodology is used to calculate the C^3 ^but the subcellular localization value is based on prediction methodology made by SUBA. For each location identified as Class D, AtPIN indicates the probability of this particular prediction to be correlated to experimental data at AtPINDB. The P_local _is a probabilistic value, thus, the higher P_local _indicates a higher probability of this particular protein been found at the predicted cellular compartment, according to the data available in AtPINDB derived from SUBA database. This posterior probability is demonstrated as:

where

exp = Experimentally demonstrated, pred = indicated by prediction and local = specific subcellular location

The last class is **Unknown: **which indicates that there is no available data to calculate the C^3 ^value or the data does not fit onto any class previously described. It is noteworthy that C^3 ^value is an active characterization due to its dependency on experimental data availability of protein interaction as well as subcellular location.

Another probability shown by AtPIN is the PEP. This is a Bayesian probabilistic score calculated based on all data available in AtPINDB so, it is dependent on the availability of experimental data. It is represented by two values, first the probability of a particular PPI be experimentally demonstrated once it was predicted, and second, same as state for the first but of both interactors were experimentally co-localized, for the release 8 those values are 2.6% and 9.0% respectively. The PEP value is unique for each AtPINDB release, an updated value is shown at website, and should be used only as a statistical evaluation of AtPINDB.

### Functional similarity weight

It has been shown in the literature that a pair of genuine interacting proteins are generally expected to have a common cellular role and proteins that have common interaction partners have a high chance of sharing a common function [31-35]. In AtPIN, due to its integrative profile, the reliability index for a reported PPI can be postulated in terms of the proportion of interaction partners that two proteins have in common. Two related mathematical approaches, CD-distance [36] and FSWeight [31], have been proposed to assess the reliability of protein interaction data based on the number of common neighbours of two proteins. Both were initially projected to predict protein functions, and lately have been shown to perform well for assessing the reliability of protein interactions [34]. Wong [37] have shown that using FSWeight, which estimates the strength of functional association, to remove unreliable interactions (low FSWeight) improves the performance of clustering algorithms.

The pairs of interacting proteins that are highly ranked by this method are likely to be true positive interacting pairs. Conversely, the pairs of proteins that are lowly ranked are likely to be false positives. The most interesting feature of the CD-distance and FSWeight is that they are able to rank the reliability of an interaction between a pair of proteins using only the topology of the interactions between that pair of proteins and their neighbors within a short radius in a graph network [32,38].

In AtPIN, we implemented the FSWeight algorithm originally proposed by Chua [31]. The functional similarity weight index on a pair of proteins A and B in an interaction graph (FSW_A, B_) is defined as:

Where

*N*_*A*_* = set of interaction partners of A; N*_*B*_* = set of interaction partners of B; λ*_*A*, *B *_*is a weight to penalize similarity weights between protein pairs when any of the proteins has too few interacting partners and is calculated as:*

where

*N*_*avg *_= *Average of interactions made by each protein in AtPINDB*.

The effectiveness of using FSWeight as a PPI reliability index was demonstrated using 19.452 interactions in yeast obtained from the GRID database [39], over 80% of the top 10% of protein interactions ranked by FSWeight have a common cellular role and over 90% of them have a common subcellular localization [32,38]. In AtPIN (release 8 of AtPINDB), using the same top 10% of protein interactions ranked by FSWeight, we show that 59% PPIs share the same sub-cellular compartment and 83% have the same function or participate in the same cellular process. A good FSWeight value threshold starting point is the top 20%, since Chua [31] and Chen [38] have demonstrated that a protein pair having a high FSWeight value, above this value, are likely to share a common function. We have made available on the AtPIN website a table with live calculation of top ranked FSWeight values ranging from the top 1% to the top 99% showing the percentage of PPIs that share the same sub-cellular localization and function, as well as the FSWeight cut off value.

### Web interface

**Figure 1:**
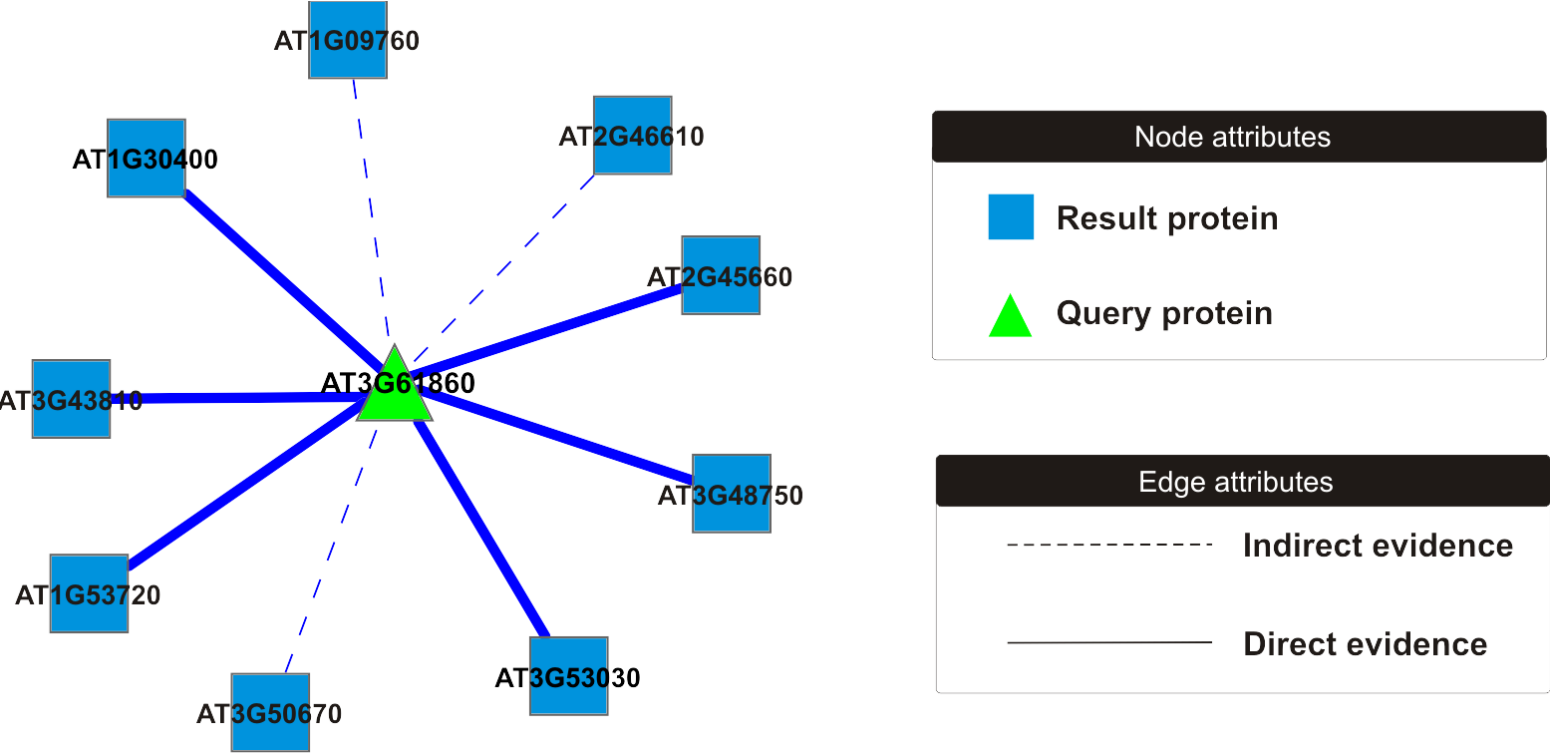
**Protein-protein interaction network generated by AtPIN export feature**. *Arabidopsis thaliana *AT3G61860 interactome through AtPIN database. The arrow thickness indicates the evidence: thin dashed line represents that it is indirect evidence and in the case of the solid thick lines it represents direct evidence.

AtPIN web interface was entirely built as a PERL script and locally hosted on a DELL Poweredge server at http://bioinfo.esalq.usp.br/atpin/. A TAIR locus name can be used to query AtPIN and the response page displays all interactions found in AtPINDB, as well as the C^3 ^value, PEP, and optionally, subcellular location information and gene ontology. The queried interactions may be visualized and manipulated online using Medusa JAVA applet [25], alternatively, the PPI network may be exported as an XGMML file to be visualized by Cytoscape. The edges shape and width indicate protein-protein interaction on the exported network, (figure 1). The thin-dashed line represents a predicted interaction and the bold line represents an experimentally-demonstrated interaction. The SIF file only represents the PPIs with no additional information. The RSP31 RNA binding protein, locus AT3G61860, was used as an example in the assembly of all the interactions in the AtPINDB. The analysis shows that RSP31 RNA binding protein interacts with nine distinct proteins, six of them being experimentally detected (Figure 1).

## Utility and discussion

We present two study cases, first encompassing the aminoacyl-tRNA synthetases (aaRS), a *de novo *experiment, and, a second found in literature, using the phytochromes proteins.

**Figure 2:**
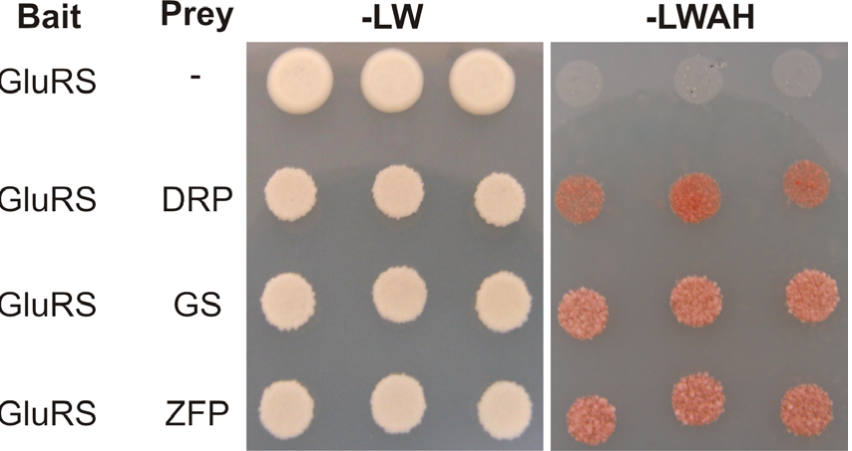
**Yeast two-hybrid confirmation of a predicted interaction**. AtGluRS interacts with a disease resistance protein (DRP), glutamine synthetase (GS) and a zinc finger protein (ZFP). Three independent AH109 transformants were spotted onto yeast minimal plates lacking leucine and tryptophan (-LW) to select for the prey and bait plasmids respectively, or lacking histidine and adenine (-LWAH) to indicate reporter gene activation. Assays use a Gal4 based yeast two-hybrid system.

The aaRS perform a crucial role in the maintenance of genetic code fidelity in all organisms. These proteins are required for catalyzing the joining of specific amino acids to their cognate tRNAs [40]. aaRS have been shown to be involved not only in protein synthesis but also in transcription, splicing, inflammation, angiogenesis and apoptosis [41]. Thus, the identification of aaRS-partner proteins may help elucidating their role in plant cells, one of our current research interests. In order to identify target proteins of *A. thaliana *cytosolic glutamyl-tRNA synthetase (gluRS) (locus AT5G26710) we combined two experimental approaches. First, analysis of the AtPIN database identified 45 candidate proteins, all of the interactions proposed by prediction analyses (Table 1). To confirm interaction of gluRS with the target proteins we performed a yeast two-hybrid system screening using At5 g26710 as a bait. Among twenty clones sequenced, the great majority was out of frame, indicating that these were false-positives. Only three sequences were in correct frame and were also found at AtPINDB (Figure 2): glutamine synthetase (AT5G35630), a zinc finger protein (AT5G24930), and a disease resistance protein (AT3G50950).

**Table 1:** Proteins identified by AtPIN as interactors with Glutamyl-tRNA Synthetase (AT5G26710)

Locus	Description
AT1G09640	Elongation factor 1B-gamma, putative/eEF-1B gamma, putative
AT1G13460	Serine/threonine protein phosphatase 2A (PP2A) regulatory subunit B', putative
AT1G29940	NRPA2 (nuclear RNA polymerase A 2); DNA-directed RNA polymerase
AT1G31070	UDP-N-acetylglucosamine pyrophosphorylase-related
AT1G52380	Ran-binding protein 1 domain-containing protein/RanBP1 domain-containing protein
AT1G52740	HTA9; DNA binding
AT1G54210	ATG12a (AUTOPHAGY 12); protein binding
AT1G57720	Elongation factor 1B-gamma, putative/eEF-1B gamma, putative
AT1G60620	ATRPAC43 (Arabidopsis thaliana RNA polymerase I subunit 43); DNA binding/DNA-directed RNA polymerase
AT1G63160	Replication factor C 40 kDa, putative
AT1G77050	DEAD/DEAH box helicase, putative
AT1G79250	Protein kinase, putative
AT1G79990	Coatomer protein complex, subunit beta 2 (beta prime), putative
AT2G17520	IRE1A (Yeast endoribonuclease/protein kinase IRE1-like gene); kinase
AT2G32850	Protein kinase family protein
AT2G35020	UTP--glucose-1-phosphate uridylyltransferase family protein
AT2G39770	CYT1 (CYTOKINESIS DEFECTIVE 1); nucleotidyltransferase
AT2G40360	Transducin family protein/WD-40 repeat family protein
AT2G45500	ATP binding
AT3G02820	Zinc knuckle (CCHC-type) family protein
AT3G10050	OMR1 (L-O-METHYLTHREONINE RESISTANT 1); L-threonine ammonia-lyase
AT3G10950	60S ribosomal protein L37a (RPL37aB)
AT3G13970	APG12/APG12B (AUTOPHAGY 12)
AT3G15970	Ran-binding protein 1 domain-containing protein/RanBP1 domain-containing protein
AT3G21700	GTP binding
AT3G26020	Serine/threonine protein phosphatase 2A (PP2A) regulatory subunit B', putative
AT3G49830	DNA helicase-related
**AT3G50950**	Disease resistance protein (CC-NBS-LRR class), putative
AT3G60240	EIF4G (EUKARYOTIC TRANSLATION INITIATION FACTOR 4G)
AT3G60245	60S ribosomal protein L37a (RPL37aC)
AT4G08500	MEKK1 (MYTOGEN ACTIVATED PROTEIN KINASE KINASE); DNA binding/kinase/kinase binding
AT4G13780	Methionine--tRNA ligase, putative/methionyl-tRNA synthetase, putative/MetRS, putative
AT4G13820	Disease resistance family protein/LRR family protein
AT5G04430	KH domain-containing protein NOVA, putative
AT5G12370	SEC10 (EXOCYST COMPLEX COMPONENT SEC10)
AT5G13520	Peptidase M1 family protein
AT5G20850	ATRAD51 (Arabidopsis thaliana Ras Associated with Diabetes protein 51); damaged DNA binding
**AT5G24930**	Zinc finger (B-box type) family protein
**AT5G35630**	GS2 (GLUTAMINE SYNTHETASE 2); glutamate-ammonia ligase
AT5G40820	ATRAD3 (ATAXIA TELANGIECTASIA-MUTATED AND RAD3-RELATED); inositol or phosphatidylinositol kinase
AT5G43430	ETFBETA; electron carrier
AT5G46190	KH domain-containing protein
AT5G54840	GTP-binding family protein
AT5G55130	CNX5 (SIRTINOL RESISTANT 1); Mo-molybdopterin cofactor sulfurase
AT5G67630	DNA helicase, putative

Bold: PPI confirmed in the yeast two-hybrid system.

Phytochromes are dimeric chromoproteins that regulate plant responses to red (R) and far-red (FR) light. Recently, Clark and co-authors [42] characterized the dimerization specificities of the *Arabidopsis *phytochromes in yeast two-hybrid analyses and by coimmunoprecipitation (co-IP), and demonstrated that two phytochrome forms, phyC (AT5G35840) and phyE (AT4G18130), do not homodimerize and, instead, heterodimerize with phyB (AT2G18790) and phyD (AT4G16250). Interestingly, the phyE heterodimeriziation with phyD was previously predicted by two different data sets present in AtPINDB and no homodimerization were predicted.

This observation shows that AtPIN might be a useful, additive and reliable strategy to map protein-protein interactions in *Arabidopsis*, once it integrates a wide range of PPIs from different sources.

## Conclusions

AtPIN is a user-friendly tool to aggregate information on *Arabidopsis thaliana *PPIs, ontology, and subcellular localization. This database may help in elucidating the intricate network of *A. thaliana *protein interactions. The AtPIN usability is aimed at new researchers as well as more skilled personnel. The XGMML and SIF file generation may help in the construction of more complex PPI networks with no previous computer language knowledge since these files can be easily merged and edited.

## Availability and requirements

The AtPIN web server is publically accessible via Http://bioinfo.esalq.usp.br/atpin. To take full advantage of the AtPIN system, a user's web browser should support AJAX and JAVA. All data downloaded from the AtPIN server are tab-delimited ASCII format.

## Authors' contributions

MMB planned, wrote and tested all the software. LLBD performed all yeast two-hybrid experiments. MCSF provided guidance during all phases of planning, designing, testing and implementing AtPIN. All authors contributed to the writing of the manuscript.
